# Ductile keratin films from deep eutectic solvent-fractionated feathers†

**DOI:** 10.1039/d1ra05123g

**Published:** 2021-08-13

**Authors:** Emmi-Maria Nuutinen, Tommi Virtanen, Raija Lantto, Mika Vähä-Nissi, Anna-Stiina Jääskeläinen

**Affiliations:** Sustainable products and materials, VTT, Technical Research Centre of Finland Tietotie 2 FI-02044 Espoo Finland emmi.nuutinen@vtt.fi +358406817126

## Abstract

Feathers, an industrial by-product, are a valuable source of keratin that could be used, for example, in the preparation of films for biomedical and packaging applications. However, the utilisation of feather keratin requires scalable processes to convert feathers into a feasible keratin stream. This paper shows how deep eutectic solvent (DES) fractionated feathers could be converted into strong films. In the DES fractionation process, two keratin fractions with different molecular weights were obtained. The films made of the high molecular weight keratin fraction had better mechanical properties and stability against moisture than the films made of the low molecular weight keratin fraction. The strength properties were further improved by cross-linking the keratin with diglycidyl ether enabling the formation of a uniform keratin network, whereas glutaraldehyde did not show a clear cross-linking effect. These keratin films could be used, for example, in food packaging or medical applications such as wound care.

## Abstract 2

In the present study, cross-linked keratin films were prepared using deep eutectic solvent (DES) fractionated feathers as a raw material.

## Graphical abstract

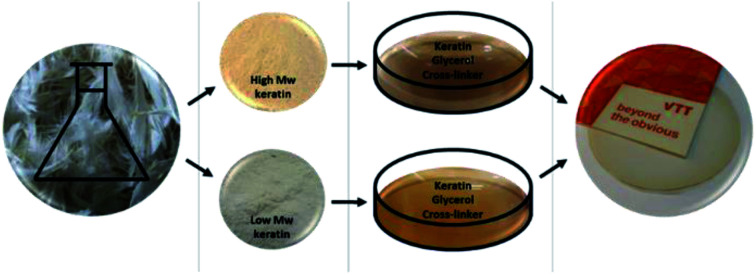

## 1. Introduction

The increased importance of environmental issues and their relation to fossil-based materials have accelerated development towards more sustainable materials during recent years. For example, the utilisation of industrial side streams in biopolymer production has attracted interest. Feathers are an abundant side stream in the poultry industry. Approximately 90% of the feathers is keratin that could be used to produce value-added applications, for example, for feed, cosmetics, electronics, agriculture, textile, composite, and medical industries.^1^ Although the native feather keratin has attractive material properties and it is abundantly available around the world year-round at low cost, at the moment, feathers are usually disposed of in landfills or reused in animal feed,^1,2^ which is a waste of material.

Feathers have a complex and hierarchical structure. Although some parts of the feathers might find applications as natural protein fibres,^3^ large-scale utilisation requires novel technologies to convert feathers into a more utilisable and homogenous form. Feather keratin has been successfully converted into for example micro- and nanoparticles, fibres, films, hydrogels, and composites.^1^ Recently, especially combining keratin with other materials has attracted attention.^4^ This type of conversion can be done using different processing methods, from which dissolution and regeneration are probably the most potential ones.^1^ More robust methods such as mechanical refining combined with an alkaline treatment^5^ and thermal processing^6^ have been used to prepare films from feathers. However, dissolving, and regenerating feathers could provide a scalable process resulting in a more uniform keratin fraction feasible for various types of products such as films.

Feather keratin is a fibrous structural protein. It is rather insoluble in common solvents due to its extensive intra- and intermolecular disulphide cross-links, crystallinity, and strong hydrogen bonding.^7^ Efficient solvents are able to disturb these interactions. The dissolution of the feather keratin is usually achieved with acid or alkaline hydrolysis, oxidation, or reduction of the disulphide bonds.^8^ The chemicals used for these reactions are often toxic, poorly recyclable, and expensive to produce. Green solvents, *N*-methylmorpholine *N*-oxide (NMMO)^9^ and ionic liquids (ILs),^10^ have been used to dissolve feathers. The green solvents are considered to possess similar physiochemical characteristics with each other such as low volatility, non-flammability, low melting point, low vapour pressure, dipolar nature, chemical, and thermal stability, high solubility, and tuneability.^11^ In addition to NMMO and ILs, a rather new type of solvents, deep eutectic solvents (DESs), have been used to dissolve feathers.^12^ DESs have similar properties with ILs and they are also easy to prepare with low preparation costs and toxicity.^13^

Aqueous solutions based on urea and 2-mercaptoethanol (2 ME),^14–17^ sodium metabisulfite (Na_2_S_2_O_5_),^18^ and cysteine^8^ as well as sodium sulphide (Na_2_S),^19–21^ have been used to solubilise feather keratin to prepare films. Solubilised feather keratin has at least partly lost its disulphide cross-linking and crystallinity, and it has a rather low molecular weight leading to films with poor mechanical properties and stability, especially in the wet state.^19,22^ The mechanical properties and the stability of feather keratin films could be improved by reforming the disulphide cross-links, increasing the chain entanglement, forming new cross-links, or incorporating reinforcing nanoparticles in the structure.^19^ New cross-links in the protein films can be formed for example with chemical cross-linkers such as glutaraldehyde^23^ and diepoxies.^24^ The mechanical properties can also be improved with plasticisers. Protein films are usually brittle. This weakness can be overcome with the plasticisers, which are typically small polyols such as glycerol, sorbitol, and polyethylene glycol.^14–16^ Plasticisers are able to disturb the hydrogen bonding and spread the protein chains apart providing flexibility.^15,16^

DESs could provide an environmentally friendly and scalable process to produce a homogenous feather keratin stream for high-value applications. In this study, feathers were fractionated with an aqueous DES composing of sodium acetate (NaOAc) and urea, and two keratin fractions of different molecular weights were obtained. These keratin fractions were evaluated for their applicability for film preparation with and without further chemical cross-linking with glutaraldehyde (GA) and 1,4-butanediol diglycidyl ether (BDE). The molecular weight and particle size distributions of the obtained keratin fractions were measured, while the film properties were studied with a focus on the evaluation of film morphology, tensile strength, water vapour permeability, solubility, swelling, and water contact angle.

## 2. Experimental

### 2.1 Materials

Feathers were supplied by Grupo SADA (Madrid, Spain) and prior to their delivery they were washed with an alkaline soap solution (95 °C for 2 h), dried (60 °C for 24 h), and then sterilised with pressurised steam (126 °C for 30 min). The absence of pathogens was confirmed with microbiological detection. The feathers were then ground into 2–15 mm pieces using an E-compactor (VTT, Finland) in which the feathers are pressed through a die using pan grinder rollers before their utilisation.

99.0–100.5% urea was purchased from Sigma-Aldrich (Germany), >99% sodium acetate anhydrous from Sigma-Aldrich (USA), glycerol (EMSURE® ACS, Reag. Ph Eur) from Merck (Germany), glutardialdehyde (25% solution in water) from Merck (Germany) and >99% 1,4-butanediol diglycidyl ether from Aldrich (USA).

### 2.2 DES fractionation & film preparation

**Fig. 1:**
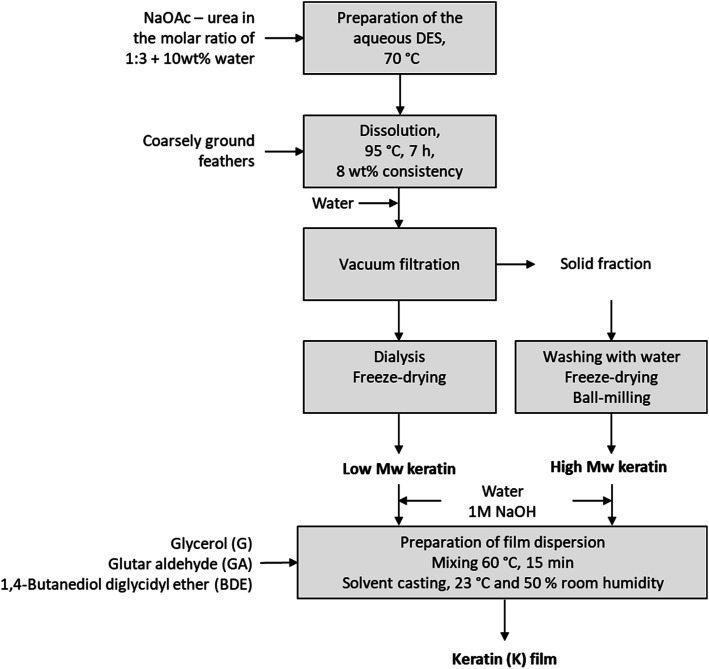
Experimental set-up for the DES fractionation and film preparation.

Fig. 1 shows the experimental set-up for the DES fractionation and the film preparation. The DES fractionation was carried out as previously described^12^ with minor modifications. In this fractionation, the DES was prepared by mixing NaOAc and urea (molar ratio 1 : 3) with 10% of water at 70 °C until a clear solution was obtained. Ground feathers (8 wt%) were added, and the solution was heated to 95 °C and kept for 7 hours under mixing. After the dissolution, water (100% on weight) was added to precipitate the keratin from the DES solution, after which the solid fraction was separated by filtration in a Buchner funnel. The precipitate was then washed with water, freeze-dried and ball-milled. The DES solution was dialysed using membranes with 3.5 kDa cut off (Spectra/Por® Standard RC Tubing, Spectrum Laboratories, CA, USA) and the dialysis was stopped when the conductivity of washing water was levelled off. The soluble keratin fraction was then collected from the dialysis tubes and freeze-dried.

The keratin films were prepared by mixing keratin (high *M*_w_ or low *M*_w_ fraction) with 50% glycerol (G) and 15 ml of water to form a dispersion. The solid content of the dispersion was 1 g, and the final concentration of glycerol in the film solution was 15 or 30 wt%. The pH of these mixtures was adjusted to 12 with 1 M NaOH solution. The dispersions were then mixed with a magnetic stirrer at 60 °C for 10 min. The mixtures were cast in either silicone or Teflon moulds depending on the analysis and dried at 23 °C and 50% relative humidity (RH) for at least 24 hours.

Glutaraldehyde (GA) was added to the mixtures in two different ways. In the first approach, the mixtures were prepared as described above with 30 wt% of glycerol, after which GA (0.1 g per 1 g of keratin) was added to the mixtures. After the GA addition, the mixtures were mixed at room temperature for 15 min and were set to dry.

In the second approach, the keratin was dispersed in water, and the pH was adjusted to 9. Then GA (0.1 g per 1 g of keratin) was added and the dispersion was stirred at 60 °C for 15 min. The pH was adjusted to 12 followed by 30 wt% glycerol addition. After mixing for 15 minutes at 60 °C for 15 min the dispersion was cast in a silicone or Teflon mould and set to dry at 23 °C 50% RH. Cross-linking with 1,4-butanediol diglycidyl ether (BDE) was carried out similarly, except that the pH was adjusted to 9.5 before adding the cross-linker.

### 2.3 Molecular weight

Matrix-assisted laser desorption ionisation time-of-flight mass spectrometry (MALDI-TOF MS) was applied to determine the molecular weight distribution of the keratin samples. Prior to the measurements, the keratin samples were dissolved in a mixture of 1.5% dithiothreitol (DTT), 0.5 M tris hydrochloride (HCl), 10% glycerol, and 2% sodium dodecyl sulphate (SDS). Sinapinic acid was selected as the matrix and dissolved to saturation in a mixture of 0.1–0.3% trifluoroacetic acid (TFA) and 50% acetonitrile. One microliter of the matrix mixture and sample was placed on the target plate and dried under air. The analysis was conducted using a Bruker mass spectrometer Autoflex II Maldi-TOF LRF50-CID (Bruker Daltonik GmbH, Germany).

### 2.4 Particle size

0.44 g of the keratin fractions were dispersed in 7.5 ml of Milli-Q water, and the pH of the dispersions was adjusted to 12 with 1 M sodium hydroxide (NaOH) solution. The particle size distribution of the low *M*_w_ keratin fraction was measured using a Zetasizer nano ZS (Malvern Instruments, Malvern, UK), and the particle size distribution of the high *M*_w_ keratin fraction was determined by laser diffraction using a Malvern Mastersizer 3000 with hydro LV liquid dispersion unit (Malvern Instruments, Worcestershire, UK). The measurement range of 0.005–5000 μm was used in the liquid module. Water was used to dilute the samples. Particle size distributions were calculated with the Fraunhofer approximation. Samples were analysed in duplicate with five parallel measurements during each run.

### 2.5 Scanning electron microscopy (SEM)

Keratin film surfaces were imaged using a Field Emission Scanning Electron Microscope (Zeiss Merlin) at an accelerating voltage of 2 kV. Samples were coated with a fine gold layer before obtaining the SEM images. Two images of each sample were taken, and the most representative images were selected for the publication.

### 2.6 Confocal laser scanning microscopy (CLSM)

The distribution of chemical components in keratin films was visualised using confocal laser scanning microscopy (CLSM) equipment consisting of a Zeiss LSM 710 (Zeiss, Jena, Germany) attached to a Zeiss Axio Imager.Z microscope. The surfaces (*xy*-direction) were imaged without cover slip as the reflection of light from diode laser line of 405 nm detected by T PMT module utilising a 20× objective (Zeiss EC Epiplan-Neofluar, numerical aperture of 0.50). For confocal imaging (*z*-direction), the films were covered with a coverslip, a diode laser line of 405 nm was used for excitation, and emission was collected at 410–491 nm. Final images were assembled of the optical sections taken using a 10× objective (Zeiss EC Epiplan-Neofluar, numerical aperture of 0.16) to the depth of 40–68 μm with 2.00 or 5.34 μm *z* step. All images were captured with a resolution of 1024 × 1024 and using ZEN software (Zeiss). At least three images were taken from each sample and the most representative ones were selected for the publication.

### 2.7 Fourier transform infrared spectroscopy (FTIR)

A Fourier transform infrared spectroscopy (FTIR) spectrometer equipped with an attenuated total reflectance (ATR) diamond crystal (Nicolet iS50, Thermo Scientific, USA) was used for the structural studies. All spectra were collected using 32 scans in a spectral range of 4000–400 cm^−1^ and with a spectral resolution of 4 cm^−1^. At least three spectra were collected from different locations of each sample and the average spectrum was calculated.

### 2.8 Nuclear magnetic resonance spectroscopy (NMR)

NMR spectra were recorded using a Bruker Avance III 500 NMR spectrometer with a magnetic flux density of 11.7 T and DMSO-d_6_ as a solvent. The spectra were acquired with a 5 mm BB(F)O double resonance probehead at 22 °C using a 30° excitation pulse, and 32 scans with a 1.5 s delay between the successive scans. Referencing was carried out using the lock frequency, and the spectra were processed using a Bruker TopSpin 4.0 and OriginPro 2020 software.

### 2.9 Mechanical properties

The mechanical properties of the keratin films were measured with a tensile tester (a Lloyd LS5 equipped with a 100N sensor, AMETEK Measurement & Calibration Technologies, Florida, USA) under the standard conditions (23 °C and 50% RH). Tensile strength was calculated by dividing the load at the break by the cross-sectional area obtained from the width (20 mm) and the thickness of the films. The film thickness was measured with a digital micrometre gauge (L&W Micrometer 51 instrument, Lorentzen & Wettre, Sweden) prior to testing. Also, Young's modulus was obtained from the measurements. Six replicate measurements for each sample were measured, and the average with standard errors were reported.

### 2.10 Water vapour permeability (WVP)

Water vapour transmission rate (g m^−2^ d^−1^) was measured with the Systech 7002 Water Vapor Permeation Analyzer (Systech Instruments Ltd, UK) at 23 °C and 50% relative humidity. The thickness of each film sample was measured using an L&W Micrometer 51 instrument prior to testing. Water vapour permeability (WVP) expressed as g m m^−2^ s^−1^ Pa^−1^ was obtained by normalising the water vapour transmission rate to the film thickness and the water vapour partial pressure gradient across the sample. Two parallel measurements were carried out for each sample.

### 2.11 Solubility and swelling

The solubility of the film samples was determined as a percentage of dry matter solubilised in Milli-Q water after 24 h immersion. Three samples with 2 cm × 3 cm dimensions of each film were cut and dried at 70 °C in an oven over night, after which they were kept in a desiccator for 15 min and weighted (*m*_0_). After the immersion in 30 ml of 25 °C Milli-Q water for 24 h, the films were again dried at 70 °C over night, kept in the desiccator for 15 min, and weighted (*m*_1_). The solubility was then calculated using the following equation:

1 Solubility (%) = 100 × (*m*_0_ − *m*_1_)/*m*_0_

The swelling was determined as a percentage of moisture content in the films after conditioning them in 75% and 90% relative humidity (RH). Three samples with 2 cm × 3 cm dimensions of each film were cut and kept in 50% RH for 24 h, after which they were weighted (*m*_50_). Then the films were conditioned in 75% RH for 24 h and weighted (*m*_75_) following conditioning in 90% RH for 24 h and weighting (*m*_90_). Temperature was kept constant at 23 °C. The swelling was calculated using the following equation:

2 Swelling (%) = 100 × (*m*_75 OR 90_ − *m*_50_)/*m*_50_

### 2.12 Water contact angle (WCA)

Contact angles for the films were determined to assess the hydrophilicity of the films. A sessile drop method with a video camera-based computer-controlled contact angle meter (Attension Theta Optical Tensiometer, Biolin Scientific, Finland) was used. A droplet volume of 6 μl (Milli-Q water) and a recording time of 120 s was used to measure the contact angle of the keratin films. The reported value is the average of recorded CAs over 120 s. An average of 2–3 replicates is reported.

## 3. Results and discussion

### 3.1 Deep eutectic solvent fractionation and film preparation

To prepare uniform films from feathers by the solvent casting method, a stable solution or dispersion made of feather keratin and volatile solvent, usually water, is required. This requires a feather treatment preferably with a solvent that is able to break the disulphide cross-links and disturb the hydrogen bonding of feather keratin.^16^ DESs are generally considered as inexpensive, relatively simple, mild, and environmentally friendly solvents to treat biomasses. In this study, feathers were fractionated using an aqueous DES composing of NaOAc and urea to obtain keratin powder to prepare the dispersions for film casting. This DES has been earlier used to process feathers^12^ but, in this study, the treatment was used on the larger scale for the first time, and two utilisable keratin fractions were successfully obtained. The first fraction was obtained by precipitation of keratin by adding water after the dissolution. The second fraction was obtained when the non-precipitated, soluble, keratin was separated from the diluted DES components by dialysis. Based on the earlier laboratory experiments, the yields of precipitated and soluble keratin fractions were about 60% and 40%, respectively.

**Fig. 2:**
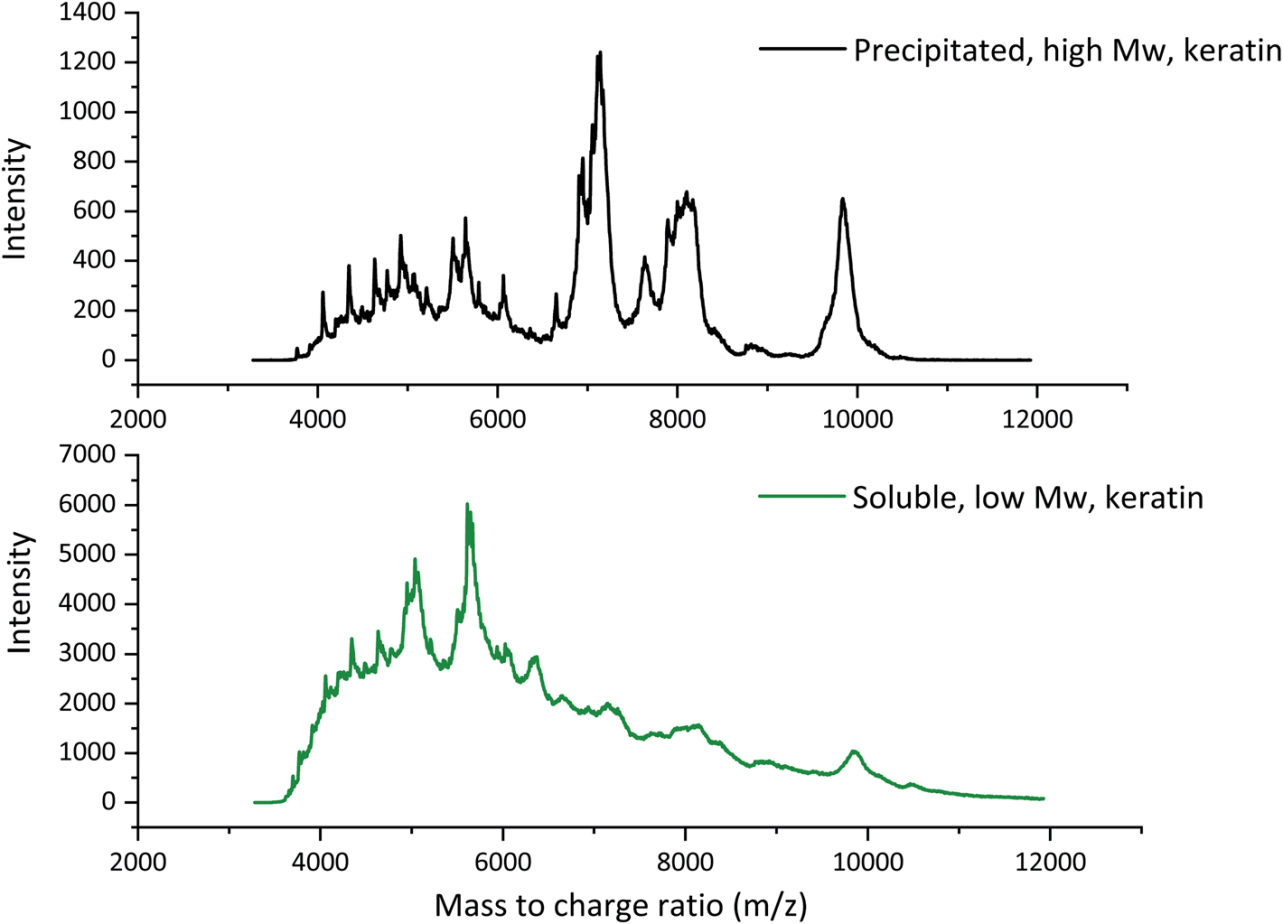
The MALDI-TOF spectra of high *M*_w_ and low *M*_w_ keratin fractions.

The molecular weights of the obtained keratin fractions were determined by MALDI-TOF MS (Fig. 2). In the precipitated keratin, the high molecular weight peaks at *ca.* 7000, 8000, and 10 000 *m*/*z* were the most intense ones, while in the soluble fraction these were present but in very low intensity. Besides these peaks, several smaller peaks were observed especially around 4000–6000 *m*/*z* indicating that the keratin fractions consisted of many different sizes of keratin fragments. Fig. 2 shows that no clear individual peaks can be seen in the soluble keratin fraction, and the spectrum is rather wide with two clearer peaks around 5000 and 5600 *m*/*z*. The higher intensities at the smaller mass to charge ratios indicate that the soluble keratin fraction consisted of small keratin fragments. It can be concluded that in the soluble keratin fraction, the keratin fragments were smaller than those in the precipitated keratin fraction. Therefore, in this paper, the fractions are referred to as low *M*_w_ keratin and high *M*_w_ keratin, respectively.

The molecular weight of native feather keratin is *ca.* 10 000 Da,^25^ which indicates that the DES fractionation cleaved also the high *M*_w_ fraction, but it still contained some un-cleaved keratin. The DES treatment disturbs the hydrogen bonding within the feather keratin, cleaves some disulphide bonds, and partly breaks down the keratin backbone.^12^ Also, the particle size distributions of the keratin dispersions at pH 12 were measured (Fig. S1†). The volume mean particle size for the high *M*_w_ keratin fraction was 24 100 ± 0.23 nm, while for the low *M*_w_ keratin fraction it was 3.43 ± 0.17 nm. With other methods like electrospraying, chemical, and mechanical treatments, average keratin particle sizes between 50 and 9200 nm have been reported.^26^

Both keratin fractions were used to prepare films by the solvent casting method. The DES-treated feather keratin fractions dispersed well in pH 12 adjusted water and allowed the formation of uniform and transparent films as the water evaporated (Fig. S2†). The films containing only the DES fractionated feather keratin were fragile, and a plasticiser had to be added to obtain films with adequate mechanical properties for sample handling. Glycerol is a common plasticiser in bio-based films. As a small, polar, and water soluble molecule which has a hydroxyl group on each carbon, glycerol is well dispersed in a protein matrix.^14^ Glycerol increases the free volume of the protein matrix thus improving the mobility and the permeability of the protein chains.^27^ For the low *M*_w_ keratin fraction, 30 wt% glycerol addition was needed, while for the high *M*_w_ fraction already 15 wt% glycerol provided films with adequate handling properties. It is thus evident that degradation of keratin to small fragments reduces its film-forming capacity.

Disulphide cross-links are a major reason for the high stability of keratin.^8^ In the DES treatment, part of these cross-links are destroyed^12^ which is detrimental for the film-forming properties. Therefore, chemical cross-linkers were applied to form new cross-links to provide additional mechanical strength and stability for the DES fractionated keratin in film applications. Glutar aldehyde (GA) and 1.4-butanediol diglycidyl ether (BDE) were selected as potential cross-linkers since they have been applied successfully for other proteins earlier,^23,28–31^ although they have not been applied for feather keratin.

GA is a well-known protein cross-linker used in for example soy protein,^23^ cottonseed protein,^28^ and gelatin^29,30^ films. GA has also been used to cross-link films made of hair keratin and cellulose.^32^ The reaction mechanism is not yet clearly understood^33^ but the cross-linking reactions most likely involve the aldehydes of GA and the amino groups of lysine.^28,33^ The cross-linking behaviour of GA depends on the pH of the solution in which the cross-linking takes place, and the cross-linking of protein with GA is usually carried out in alkaline conditions. In alkaline conditions, dialdehyde condensate and the amount of α,β-unsaturated polymers increase.^28^ The higher the pH is, the faster the polymerisation.^28,34^

BDE was used as an alternative cross-linker for the keratin fractions. BDE has low-toxicity^31^ compared to GA^29^ and would therefore be more preferred for film applications. BDE has been previously used to cross-link films made of gelatin.^31^ Also, other diepoxies have been used to cross-link protein films.^24,35,36^ Soy proteins films have been cross-linked with 1,2,3-propanetriol diglycidyl ether^36^ and ethylene glycol diglycidyl ether,^35^ while Tanabe *et al.*^24^ cross-linked wool keratin films with ethylene glycol diglycidyl ether and glycerol diglycidyl ether. When diepoxy is used to cross-link the proteins in alkaline conditions, the reaction takes place between the epoxy and the amino groups, and it involves the ring-opening of the epoxy.^31,36^

### 3.2 Keratin film characterisation

#### 3.2.1 Morphology

**Fig. 3:**
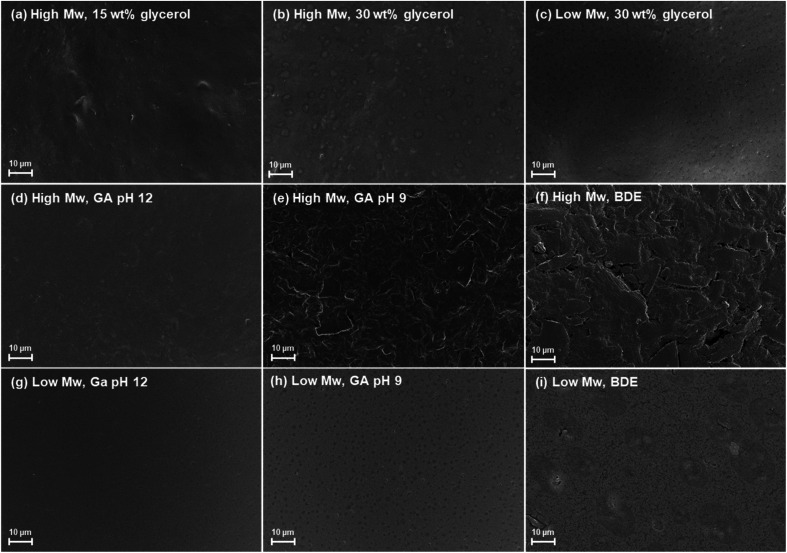
SEM images of the films made of the high *M*_w_ keratin fraction with (a) 15 and (b) 30 wt% glycerol concentrations, the (c) film made of low *M*_w_ keratin fraction with 30 wt% glycerol, and the films made of the (d–f) high and (g–i) low *M*_w_ keratin fractions and plasticised with 30 wt% glycerol and cross-linked with glutar aldehyde (GA) at pH (d and g) 12 and (e and h) 9 as well as with (f and i) 1.4-butanediol diglycidyl ether (BDE) at pH 9.5.

SEM and CLSM were used to characterise the morphology of the films. Fig. 3a shows that the keratin film plasticised using 15 wt% glycerol concentration, was homogenous with some minor inhomogeneity but no clear phase separation. Glycerol is known to increase the homogeneity of the feather keratin film surface compared to films without this plasticiser.^14,20^ The particles shown in Fig. 3a are aggregated keratin particles. Fig. 3b and c show the keratin films made of the high and low *M*_w_ keratin fractions plasticised with 30 wt% glycerol. Compared to the films plasticised with the lower glycerol concentration, these films showed more inhomogeneity (Fig. 3b) which could indicate phase separation. Glycerol disperses evenly in the protein matrix. However, due to its small size, it is also prone to migrate onto the film surface.^37^ In addition to the inhomogeneity, Fig. 3c shows small cracks on the surface. Cracking typically occurs in the drying process when the particles consolidate as the solvent leaves the dispersion.^38^ Cracking on the film surface made of the low *M*_w_ keratin fraction can be also observed with a CLSM (Fig. S3a†). The cracking of the low *M*_w_ keratin fraction may be related to the lower molecular weight compared to the high *M*_w_ keratin fraction, making the films more fragile. With the CLSM, all the films show some structural inhomogeneity on their surfaces (Fig. S3a–c†), which may indicate the phase separation due to the glycerol migration. Permeable imaging with CLSM shows a rather homogenous film structure for the films made of the low *M*_w_ keratin fraction (Fig. S3d†), while the films made of the high *M*_w_ keratin contained keratin particle aggregates (Fig. S3e and f†). This could be explained by the different particle sizes in the dispersions.

Fig. 3d–f and g–i show the cross-linked keratin films. When films in Fig. 3d–f are compared to film in Fig. 3b, which is the film before cross-linking, based on surface appearance, changes are evident. The morphology of the film surfaces is clearly rougher when cross-linking of the high *M*_w_ keratin fraction was carried out with GA at pH 9 and with BDE indicating the rearrangement of keratin chains (Fig. 3b and e–f). On the other hand, Fig. 3d shows that when the high *M*_w_ keratin fraction was cross-linked with GA at pH 12, the film surface becomes smoother. This can also be observed with the low *M*_w_ keratin fraction (Fig. 3g). The smoothness appearing on the surface may result from the polymerisation of GA. When the low *M*_w_ keratin fraction was cross-linked with GA at pH 9 (Fig. 3h), no major changes in the morphology can be observed when compared to non-cross-linked film (Fig. 3c), while with BDE (Fig. 3i) changes towards a more heterogeneous surface structure can be observed.

#### 3.2.2 Molecular structure

The chemical structures of the films were analysed by ^1^H-NMR and ATR-FTIR. ^1^H-NMR was carried out only to the films made of the low *M*_w_ keratin fraction as they were soluble in DMSO. In the ^1^H-NMR spectra, the cross-linked films are shown together with the films which are not cross-linked but plasticised with glycerol (Fig. S4†). ^1^H-NMR spectra (Fig. S4†) are typical for keratin^39^ with glycerol.^40^ In all the spectra, a peak at about 4.5 ppm can be assigned to the protons in the OH groups of glycerol and the group of intense peaks centred at about 3.4 ppm can be assigned to the protons in the CH_2_ and CH groups of glycerol.^40^ Fig. S4† shows that in the film cross-linked with GA at pH 12, the intensity and shape of these peaks change indicating changes in the glycerol structure. These changes are not seen when the keratin films were cross-linked with GA at pH 9 or with BDE. When the keratin fractions were cross-linked with GA at pH 12, glycerol was added before the cross-linker, while with GA at pH 9 and with BDE, the glycerol was added after the cross-linker. Thus, reactions between GA and glycerol might have taken place instead of keratin, which could explain the changes in the glycerol structure together with the effect of different pH. In the BDE cross-linked spectrum (Fig. S4†), changes around 7.00–6.50 ppm can be observed, especially the intensity decrease of the peak at 6.98 ppm. In this region, peaks assigned for the amide protons of the glycine amide, asparagine, and glutamine residues can be observed.^41^ Thus, indicating the possible reaction of amides with BDE. Besides the cross-linking, no major changes in the keratin chemical structure took place during the cross-linking according to the NMR spectra.

ATR-FTIR spectra were measured for all the film samples as well as for both keratin fractions (Fig. S5†). These spectra are typical for keratin,^20^ and similar spectra were obtained for DES fractionated feather keratin also earlier.^12^ Like ^1^H-NMR indicated, no major changes in the keratin structure can be observed when keratin was used to prepare films or cross-linked. In all the film samples, new bands at 1098, 1042, and 995 cm^−1^ are due to glycerol in the films.^40^ Also, an increase in intensities in the area of 3000–3500 cm^−1^ (O–H stretching and N–H bending) and 2870–2970 cm^−1^ (C–H stretching) bands can be seen in all the film samples after the glycerol addition.^40^ In the samples, which were cross-linked with GA at pH 12, an increase in the 2850 and 2915 cm^−1^ bands can be observed, which are typical band positions for C-stretching vibrations in alkanes. This could indicate polymerisation of GA or glycerol prior to its cross-linking with keratin. Simultaneously, in the samples which were cross-linked with BDE, the relative intensity ratio between amide II band at 1480–1570 cm^−1^ (NH bending and CH stretching vibration) and amide I band at 1600–1700 cm^−1^ (C=O stretching) increased compared to other samples, which may be due amine vibration changes after the cross-linking.

#### 3.2.3 Mechanical properties

**Fig. 4:**
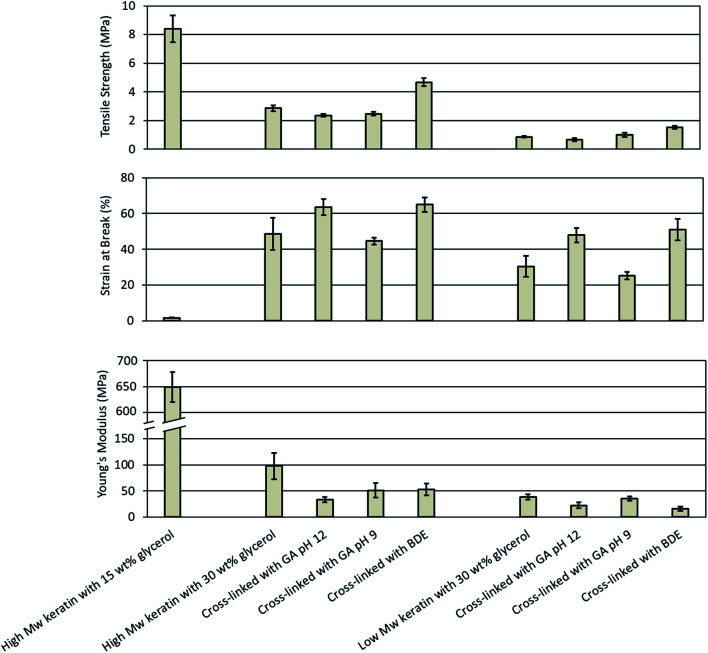
Mechanical properties of the keratin films made of the high and low *M*_w_ keratin fractions plasticised with glycerol and cross-linked with glutaraldehyde (GA) at pH 12 and 9 as well as with 1.4-butanediol diglycidyl ether (BDE) at pH 9.5.

Fig. 4 shows the tensile strength, the Young's modulus, and the strain at break for the keratin films, and examples of stress–strain curves are presented in Fig. S6.† When the glycerol concentration of the films was increased from 15 to 30 wt%, the tensile strength decreased by 65% from 8.4 ± 1.0 MPa to 2.9 ± 0.2 MPa and the Young's modulus by 85% from 649 ± 29 MPa to 98 ± 25 MPa, while the strain at break increased by 2765% from 1.7 ± 0.2% to 48.7 ± 9.0%. This can be explained by an increased free volume in the keratin matrix as the internal hydrogen bonding reduces. The films made of the high *M*_w_ keratin fraction after the glycerol addition are comparable with the literature. In the study of Moore *et al.*,^14^ the tensile strength decreased by 88% from 16.6 ± 5.5 MPa to 2.0 ± 0.2 MPa while the strain at break increased by 1777% from 1.7 ± 0.2% to 31.9 ± 4.5% after a 0.09 g glycerol per g keratin addition.

In this study, the films made of the low *M*_w_ keratin fraction with 15 wt% glycerol concentration were too fragile to handle and therefore only the results for the films with 30 wt% glycerol concentration were analysed. The tensile strength, the Young's modulus, and the strain at break for the films made of the low *M*_w_ keratin fraction were 0.9 ± 0.1 MPa, 38 ± 5 MPa, and 30.4 ± 5.8%, respectively. The poorer mechanical properties of the films made of the low *M*_w_ keratin fraction were as expected due to the substantially lower molecular weight compared to the high *M*_w_ keratin fraction.

Cross-linking keratin resulted in a substantial increase in mechanical properties of the films. When GA was used to cross-link the keratin films at pH 12, an increase in the strain at break and a decrease in the stiffness was observed, while there was no notable difference in the tensile strength (Fig. 4). The strain at break increased by 30% and 58% from 48.7 ± 9.0% and 30.4 ± 5.8% to 63.5 ± 4.5% and 47.9 ± 4.1% and the Young's modulus decreased by 66% and 42% from 98 ± 25 MPa and 38 ± 5 MPa to 33 ± 5 MPa and 22 ± 6 MPa for the films made of the high and low *M*_w_ keratin fractions, respectively. The increase in the strain at break and the decrease in the stiffness are characteristic of the plasticising effect. From the ^1^H-NMR and ATR-FTIR data, it was concluded that changes in the glycerol structure and polymerisation took place when the films were cross-linked with GA at pH 12. However, when the cross-linking was carried out with GA at pH 9, no significant changes in the mechanical properties were observed (Fig. 4). In a previous study, it was observed that GA provided a slight increase in the tensile strength and the Young's modulus for the feather keratin films in a wet state.^19^ A GA addition has also been reported to improve the tensile strength and the strain at break of soy protein films^23^ as well as the tensile strength and the Young's modulus of gelatin films.^29^ However, in this study, the GA addition did not improve the strength properties, which may indicate that new cross-links neither formed nor were they enough to improve the tensile strength. It is suggested that GA reacts with the amino groups of lysine,^28,33^ and the content of lysine in feather keratin is low,^9^ which could explain the low degree of cross-linking.

BDE was used to cross-link keratin films as an alternative to GA. Fig. 4 depicts that cross-linking with BDE was superior in improving the tensile strength of the films compared to GA. BDE improved both the tensile strength as well as the strain at break, while in the Young's modulus a small decrease was observed (Fig. 4). After the BDE addition the tensile strength of the films made of the high *M*_w_ keratin fraction increased by 62% from 2.9 ± 0.2 MPa to 4.7 ± 0.3 MPa and the strain at break by 33% from 48.7 ± 9.0% to 65.0 ± 4.0%, while the Young's modulus decreased by 46% from 98 ± 25 MPa to 53 ± 11 MPa. Fig. 4 shows that in the films made of the low *M*_w_ keratin fraction, the tensile strength increased by 67% from 0.9 ± 0.1 MPa to 1.5 ± 0.1 MPa, the strain at break by 68% from 30.4 ± 5.8% to 51.0 ± 6.0% and the Young's modulus decreased by 58% from 38 ± 5 MPa to 16 ± 4 MPa. New cross-links between the cross-linker and the keratin fractions were most probably the reason for the increased strength supporting the data obtained from ^1^H-NMR. The improvements in the strain at break could be explained by the plasticising effect of the secondary hydroxyl groups and hydroxyl-terminated pendant groups from the hydrolysed un-reacted epoxides.^31^ In a previous study, when BDE was added to gelatine films, only an improvement in the strain at break was observed, and this improvement was explained with the plasticising effect of BDE.^31^ Tanabe *et al.*^24^ used ethylene glycol diglycidyl ether (EGDE) and glycerol diglycidyl ether (GDE) to cross-link the extracted wool keratin. The films made only from keratin were too fragile to handle, while after the cross-linker addition, they were able to reach values of 23 ± 6 MPa for the ultimate strength, 12 ± 6% for the ultimate elongation, and 372 ± 235 MPa for the Young's modulus.^24^

#### 3.2.4 Water vapour permeability

**Fig. 5:**
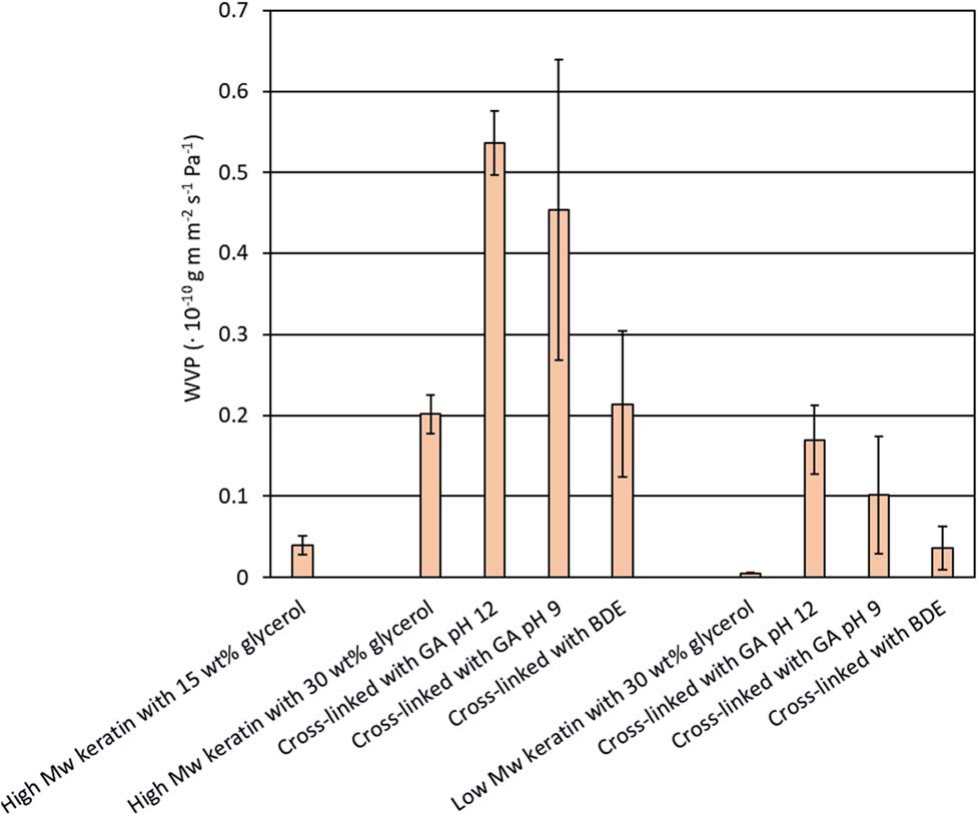
Water vapour permeability (WVP) of the keratin films made of the high and low *M*_w_ keratin fractions, plasticised with glycerol and cross-linked with glutaraldehyde (GA) at pH 12 and 9 as well as with 1.4-butanediol diglycidyl ether (BDE) at pH 9.5.

Biodegradable biomaterials are typically sensitive to moisture and their properties change when the relative humidity changes. Protein films generally have a high water vapour permeability (WVP) but a low gas permeability.^42^ The permeability properties depend on many factors, such as the ratio between the crystalline and amorphous regions, the chain mobility, and the interactions between the polymers and the permeating gases.^30^Fig. 5 shows the WVP values obtained for the keratin films. When the glycerol concentration of the high *M*_w_ keratin films was increased from 15 wt% to 30 wt%, the WVP value increased by 400% from 0.04 × 10^−10^ ± 0.01 × 10^−10^ g m m^−2^ s^−1^ Pa^−1^ to 0.20 × 10^−10^ ± 0.02 × 10^−10^ g m m^−2^ s^−1^ Pa^−1^. A similar effect has been previously reported *i.e.* by Martelli *et al.*^15^ Glycerol is a small molecule with hydroxyl groups, and as its concentration in the films increase, the hydrophilicity, as well as the mobility and the free volume between the polymer chains, increase making the films more prone to the water adsorption.^15^Fig. 5 shows that the WVP value of the films made of the low *M*_w_ keratin fraction was 0.005 × 10^−10^ ± 0.0003 × 10^−10^ g m m^−2^ s^−1^ Pa^−1^ which is lower compared the films made of the high *M*_w_ keratin fraction. A denser molecule matrix decreases the permeability.^30^ The low *M*_w_ keratin fraction obtained from the DES fractionation has a smaller molecular weight than the high *M*_w_ keratin fraction, which could allow the tighter packaging of the keratin fragments in the films giving the lower WVP value.

After the cross-linking, no improvement in the WVP values was obtained, which was unexpected (Fig. 5). An increase in the WVP values of the cross-linked keratin films was noticed when the cross-linking was carried out with GA, while with BDE no significant changes were observed (Fig. 5). It has been previously reported that chemical cross-linking can improve the WVP of protein films by making the molecule matrix denser.^30,31^ However, similar behaviour has also been reported with GA cross-linked whey protein films.^43^ Ustunol & Mert^43^ suggested that the increase in WVP values might be due to the additional polar groups in the film structure due to cross-linkers and the cross-linking reactions. Furthermore, in this study, no clear indications of successful GA cross-linking were observed. Another explanation for the increased WVP values could be the accumulation of water molecules in the structure.^43^ Water can act as a plasticiser increasing the mobility of the keratin molecules and further facilitate the diffusion of water molecules.^19,43^ SEM images also showed that the keratin films were not homogenous and cracking of the film surface was observed. This could explain the increased WVP values after the cross-linking and the significant dispersion between some of the replicates. However, the WVP values obtained for the films made of the DES fractionated keratin are lower than previously reported glycerol plasticised feather keratin films (35.5 × 10^−10^ g m m^−2^ s^−1^ Pa^−1^ (ref. 44) and 3.5 × 10^−10^ g m m^−2^ s^−1^ Pa^−1^ (ref. 45)). WVP values of a GA cross-linked gelatin film (0.094 × 10^−10^ g m m^−2^ s^−1^ Pa^−1^ (ref. 30)) and a BDE cross-linked gelatin film (0.197 × 10^−10^ g m m^−2^ s^−1^ Pa^−1^ (ref. 31)) are in a similar range with this study.

#### 3.2.5 Solubility, swelling and contact angle

Due to the hydrophilic nature of protein films, they are typically sensitive to water. When immersed in water, films made of the DES fractionated keratin disintegrated. Only the cross-linked films made of the high *M*_w_ keratin fraction remained as continuous films that could be analysed further and therefore, only the results from the cross-linked samples are reported. From Table 1, it can be seen that approximately 40% of the film weight disintegrated in water. Cross-linking is known to reduce the solubility of feather keratin^45^ and other protein films^30,43^ into water. It is suggested that in the cross-linked network, proteins interact less with water molecules.^30^ The data obtained from ^1^H-NMR and the mechanical testing indicated that the cross-linking with BDE might have taken place, while no clear evidence of the cross-linking with GA has been observed. However, all the used methods indicated that some changes took also place after the GA addition, which could explain that the films remained intact. The films in this study contained 30 wt% of glycerol as the plasticiser, which means that not only small molecular glycerol exudated out of the films. From the MALDI-TOF MS data, it was seen the DES fractionated keratin consisted of many different sizes of keratin fragments. Thus, it is likely that the keratin fragments with the lower molecular weight dissolved from the film structure. Moreover, it seems that the molecular weight of the low *M*_w_ keratin fraction is so low that the cross-linkers were unable to form a keratin network that would be stable enough to remain intact when immersed in water.

**Table 1:** Solubility, swelling, and water contact angles for the glycerol plasticised keratin films prepared from the high *M*_w_ keratin fraction with and without cross-linking. The films are cross-linked with glutar aldehyde (GA) at pH 12 and pH 9 and with 1.4-butanediol diglycidyl ether (BDE) at pH 9.5

Sample	Solubility (%)	Swelling (%)		Contact angle (°)
		75% RH	90% RH	
15 wt% glycerol	—	19.71 ± 9.96	23.00 ± 0.66	45.84 ± 4.57
30 wt% glycerol	—	5.90 ± 5.55	32.73 ± 0.86	60.97 ± 0.34
Cross-linked with GA pH 9	45.14 ± 2.38	4.70 ± 8.14	20.71 ± 11.61	62.88 ± 8.97
Cross-linked with GA pH 12	38.43 ± 1.87	10.36 ± 8.77	15.26 ± 6.67	60.90 ± 8.36
Cross-linked with BDE	42.32 ± 6.32	8.84 ± 0.45	21.42 ± 0.47	90.95 ± 0.32

Protein films are sensitive to the changes in the relative humidity as water acts as a strong plasticiser in natural polymer films by accumulating in the structure.^45^ Thus, the swelling of the films was measured in different humidities (Table 1). While the solubility was measured only for the cross-linked films made of the high *M*_w_ keratin fraction, the swelling could be measured for the films made of the high *M*_w_ keratin fraction with and without the cross-linking. The films made of the low *M*_w_ keratin fraction became soft or gel-like to remain intact during the measurement. The swelling was measured at 75% and 90% relative humidities. The swelling of the films was followed by the increased humidity (Table 1). A significant dispersion in the results indicates that the films are not uniform. The only exception is the films cross-linked with BDE. These films show smaller scatter between the parallel samples which could indicate that BDE was able to form a more uniform cross-linked keratin network compared to the GA cross-linked films and the films without the cross-linker. This result supports also the other used methods in which indications of the successful cross-linking with BDE have been observed. At 90% relative humidity the BDE cross-linked film swelled only 21.42 ± 0.47% while the films without the cross-linking swelled 32.73 ± 0.86%. Also, in the study of Martucci *et al.*,^31^ the swelling of gelatin films decreased from 18% to 11% when gelatin was cross-linked with BDE. It is speculated that the cross-linking forms a network in which the hydrophilic groups in the protein are not available for water sorption, which could then decrease the moisture content of the films. However, at the same time, BDE contains hydroxyl groups that can bind water.^45^

The wettability and especially the surface properties of the keratin films were investigated with the water contact angle (WCA) measurements. Again, only the films made of the high *M*_w_ keratin fraction were measured as the water-resistance of the films made of the low *M*_w_ keratin fraction was too poor. The results are reported in Table 1. All the samples, except the films cross-linked with BDE, had a WCA value below 90°, indicating that the surfaces of the films were wetted. After the DES fractionation, feather keratin was precipitated using water causing its hydrophilic groups to be exposed to the surface.^46^ When the WCA is over 90°, the film can be considered hydrophobic. The films cross-linked with BDE showed hydrophobicity with low scattering between the parallel samples indicating that the addition of BDE formed a more uniform surface in which hydrophilic groups are not exposed. This result is, again, in a line with the other results, especially with the swelling data.

## 4. Conclusions

An environmentally friendly and scalable treatment to produce homogenous feather keratin fractions, which could be further used to prepare films was demonstrated. An aqueous, inexpensive, and food-grade DES was used to treat the feathers, and two feasible keratin fractions with different molecular weights were obtained. Both obtained fractions were successfully used to prepare films by a solvent casting method, and the molecular weight of keratin had a determining effect on the success and properties of the film. A plasticiser addition was needed to keep the films intact, and for the lower molecular weight keratin fraction, the need for the plasticiser was higher. The higher molecular weight keratin fraction had better mechanical properties and was more stable against moisture, while the lower molecular weight keratin fraction provided better water vapour permeability. Two established protein cross-linkers, glutar aldehyde (GA) and 1,4-butanediol diglycidyl ether (BDE), were tested to modify the film properties. BDE worked well in the cross-linking of feather keratin, unlike GA. Both cross-linkers showed changes in the film properties without breaking the keratin structure, but with BDE clear indications of the formation of new covalent bonds and a uniform keratin network were observed. The BDE addition improved the mechanical properties and the stability against moisture of the films. It can be concluded that considering the film properties, a dense keratin network and a high molecular weight of keratin are recommended but they can also be controlled by cross-linking. These types of protein films can be used, for example, in food packaging or medical applications such as wound care.

## Conflicts of interest

There are no conflicts to declare.

## Supplementary Material

RA-011-D1RA05123G-s001
