# Supplementary material for: Ductile keratin films from deep eutectic solvent-fractionated feathers
Source: RSC Adv. 2021 Aug 13;11(44):27512–22. doi: 10.1039/d1ra05123g (PMC9037859; doi:10.1039/d1ra05123g)
Supplement: RA-011-D1RA05123G-s001 [file RA-011-D1RA05123G-s001.pdf]

Electronic Supplementary Material (ESI) for RSC Advances.
This journal is © The Royal Society of Chemistry 2021

Electronic supplementary information (ESI)

Ductile keratin films from deep eutectic solvent-fractionated feathers

Authors: Emmi-Maria Nuutinen*[†], Tommi Virtanen[†], Raija Lantto[†], Mika Vähä-Nissi[†], Anna-Stiina Jääskeläinen[†, ‡]

† Solutions for Natural Resources and Environment, VTT, Technical Research Centre of Finland, Tietotie 2, FI-02044, Espoo, Finland

‡ Present address: Kemira Oyj, R&D and Techology, Luoteisrinne 2, 02270, Espoo, Finland

*Corresponding author: E-mail: emmi.nuutinen@vtt.fi. Telephone: +358406817126.

3.1 Deep eutectic solvent fractionation and film preparation

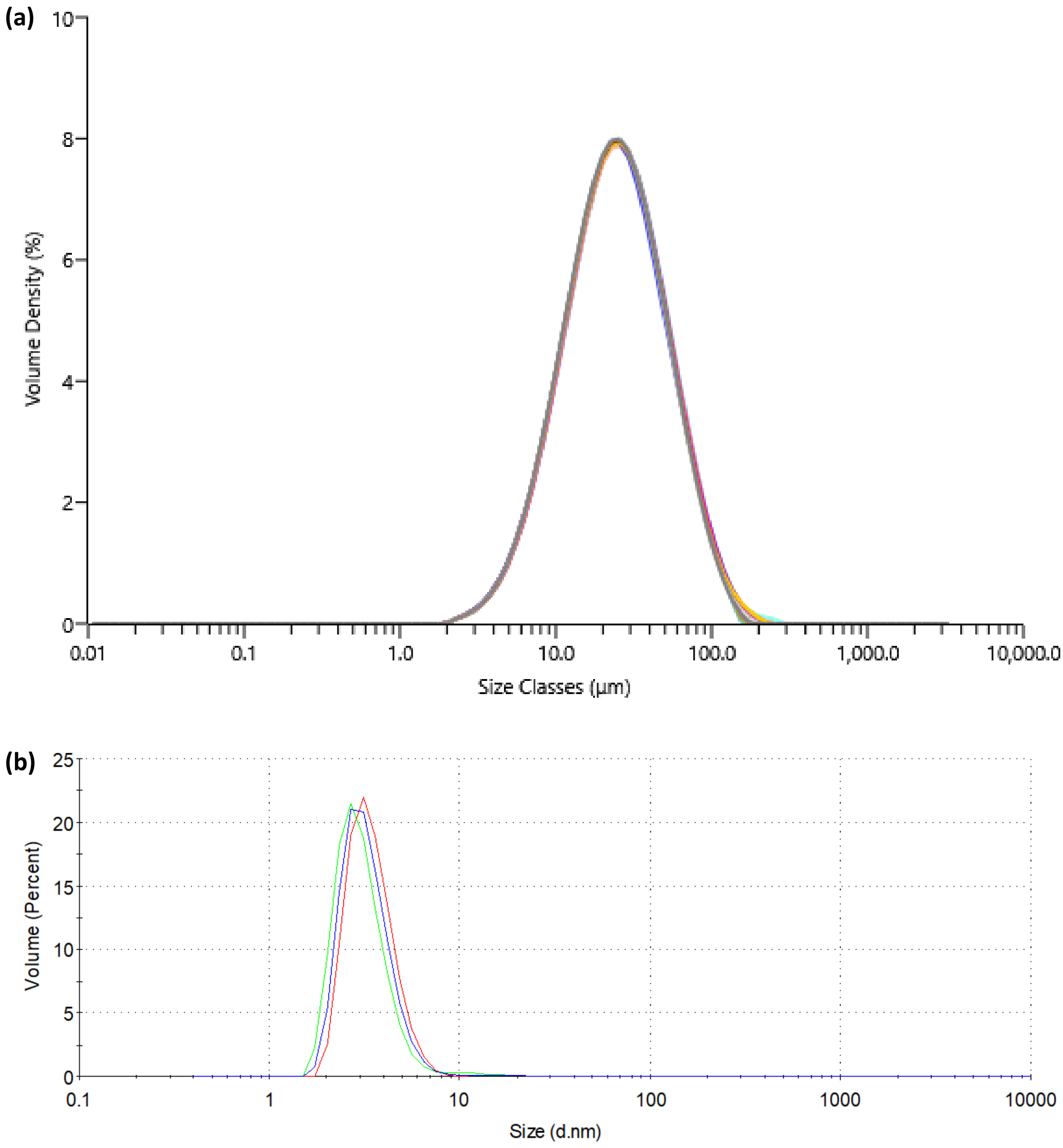

Fig. S1 Mean particle sizes of the (a) high and (b) low Mw keratin fractions.

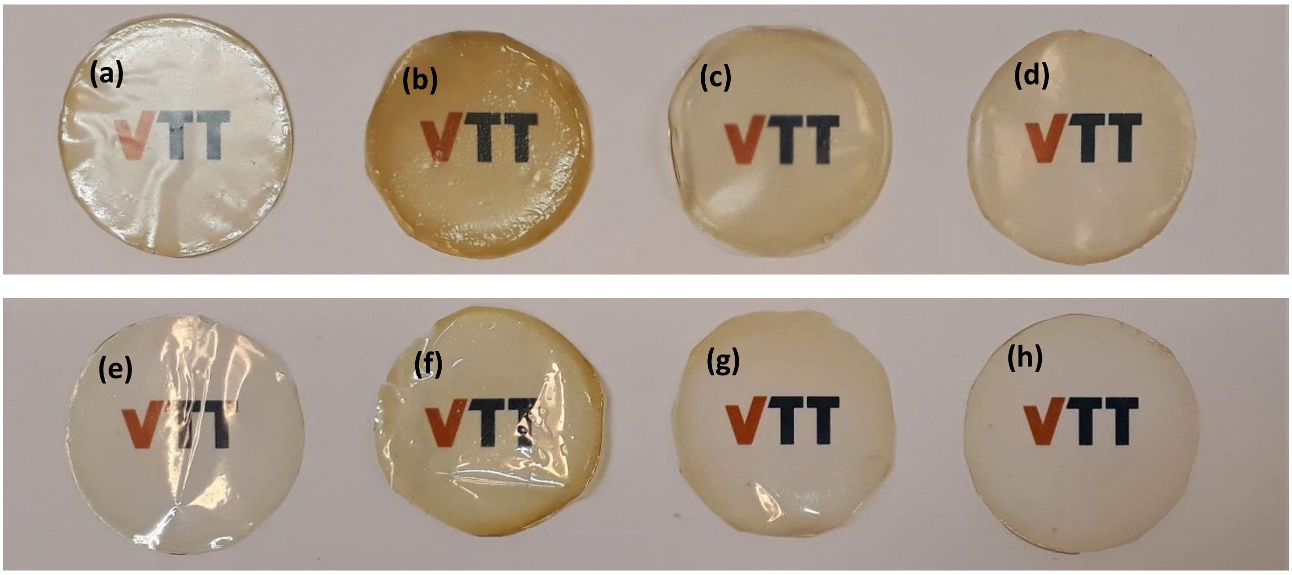

Fig. S2 Glycerol plasticised keratin films made of (a) high and (e) low Mw keratin fractions and which are cross-linked with glutaraldehyde at (b,f) pH 9 and (c,g) 12 as well as with (d,h) 1.4-Butanediol diglycidyl ether at pH 9.5.

3.2.1 Film morphology

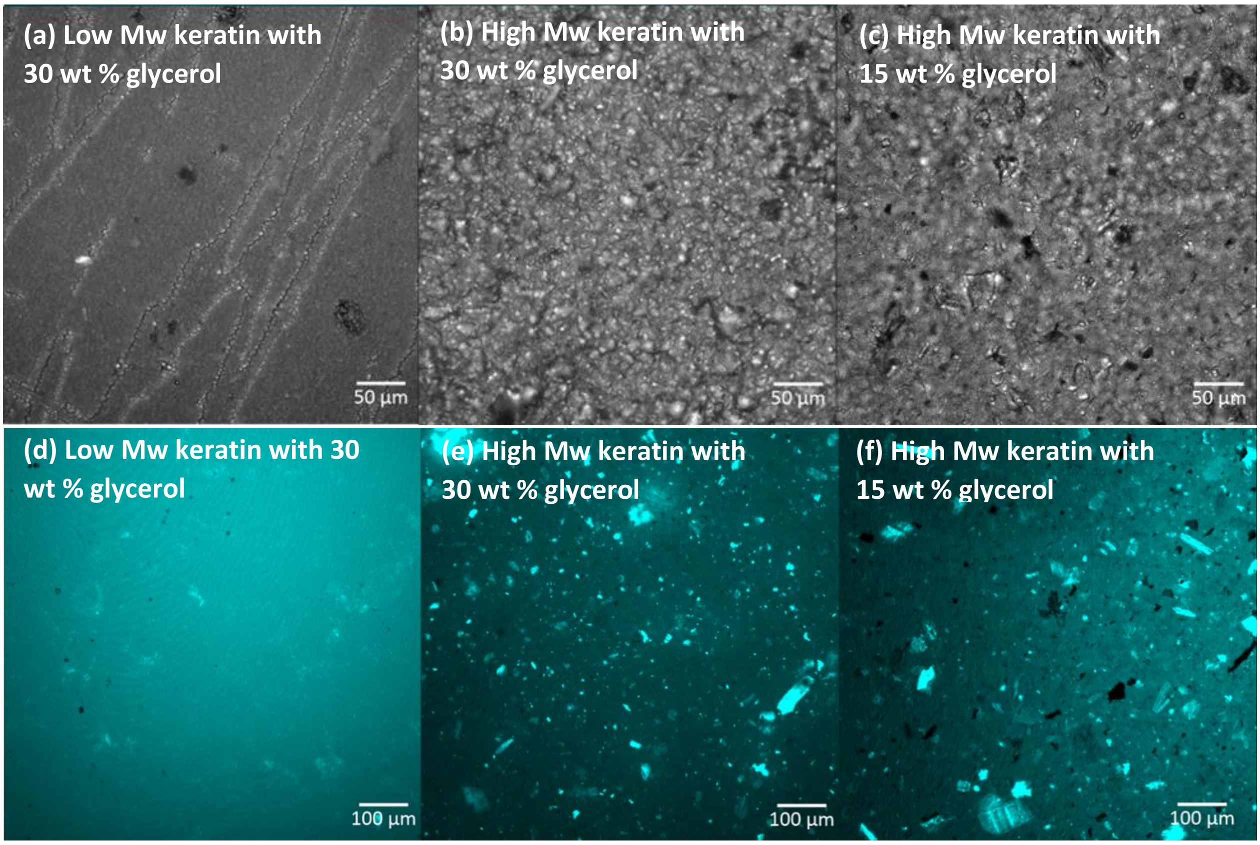

Fig. S3 Optical microscopy images of the glycerol plasticised keratin films from the surface (a-c) and permeable imaging (d-f). The films are made of the low and high Mw keratin fractions and plasticised with glycerol.

3.2.2 Molecular structure

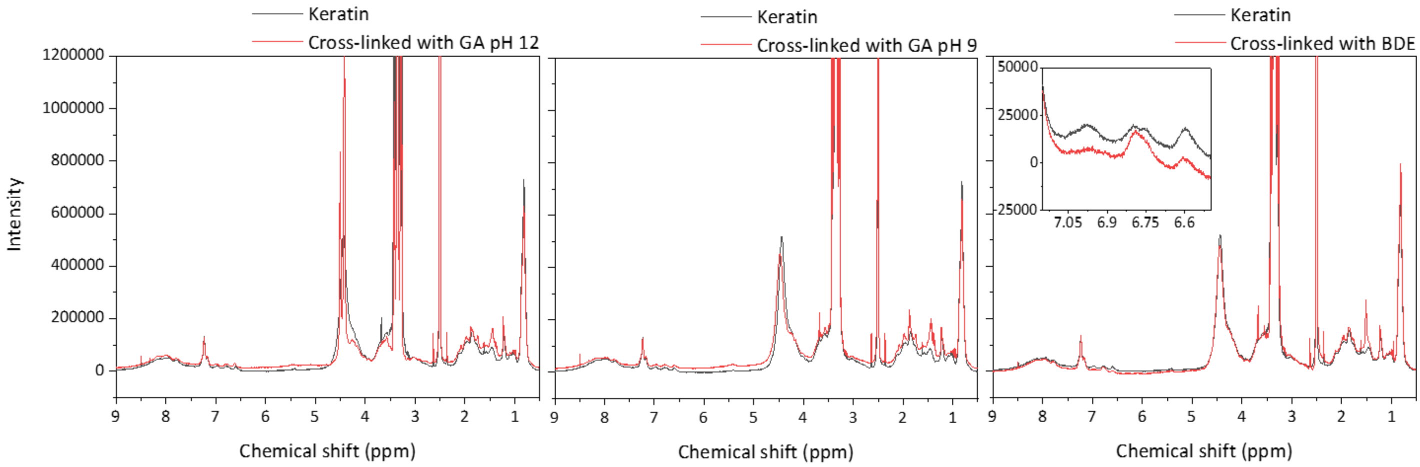

Fig. S4 ^{1}H-NMR spectra of the low Mw keratin fraction, the films made of this fraction and plasticised with 30 wt% glycerol concentration and cross-linked with glutaraldehyde (GA) at pH 12 and 9 as well as with 1.4-butanediol diglycidyl ether (BDE) at pH 9.5.

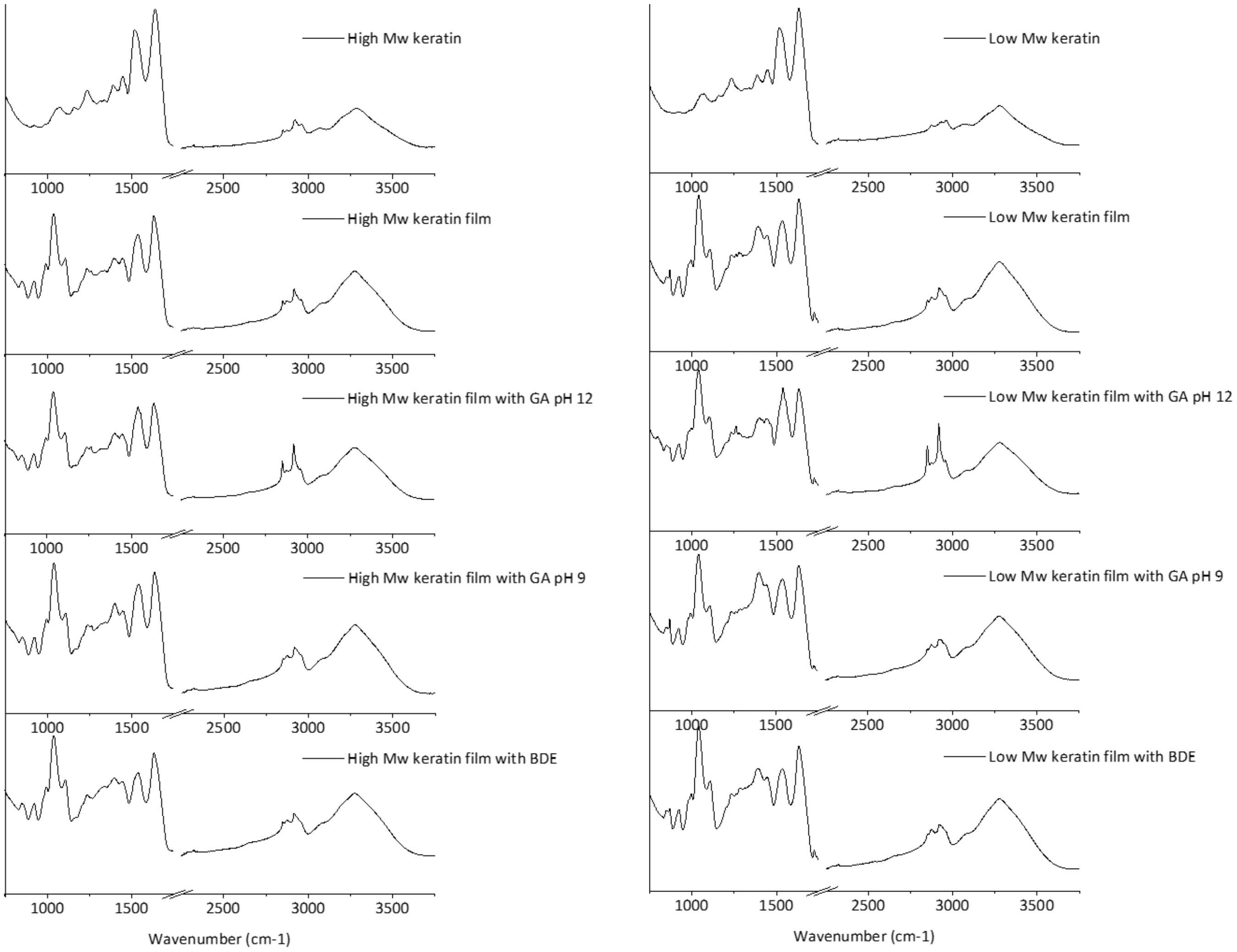

Fig. S5 ATR-FTIR spectra of the high and low Mw keratin fractions, the films made of these fractions and plasticised with 30 wt% glycerol concentration and cross-linked with glutar aldehyde (GA) at pH 12 and 9 as well as with 1.4-butanediol diglycidyl ether (BDE) at pH 9.5.

3.2.3 Mechanical properties

(a)

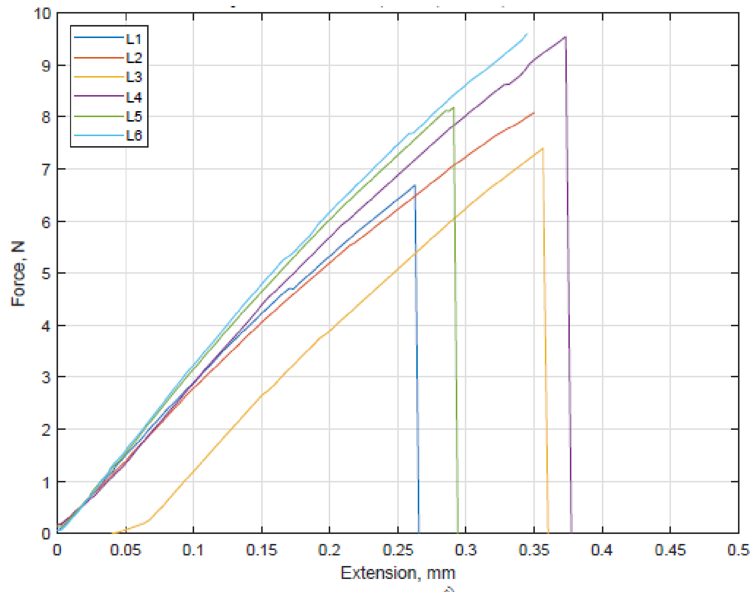

	Thickness (mm)	Young's Modulus (MPa)	Tensile Strength (MPa)	Load at Maximum Load (N)	Extension at Maximum Load (mm)	Load at Break (N)	Extension at Break (mm)	Stress at Break (MPa)	Strain at Break	Stiffness (N/m)	Work to Break (Nmm)
Ave	65,8	649	8,39	8,28	0,33	8,28	0,33	8,39	0,0166	32008	1,45
STD	4,7	29	0,95	1,06	0,04	1,06	0,04	0,95	0,0020	2183	0,34

Treg15gly Speed 2 mm/min, strip width 15 mm, gauge 20 mm, load cell 100 N

(b)

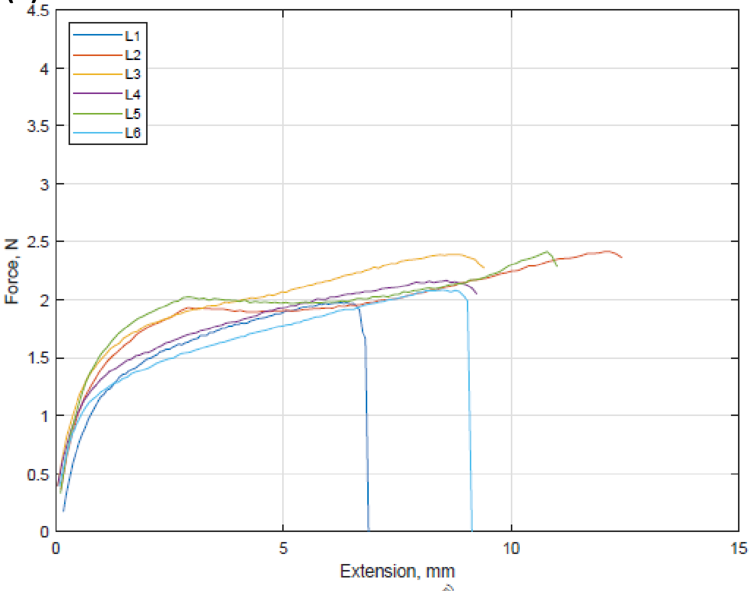

	Thickness (mm)	Young's Modulus (MPa)	Tensile Strength (MPa)	Load at Maximum Load (N)	Extension at Maximum Load (mm)	Load at Break (N)	Extension at Break (mm)	Stress at Break (MPa)	Strain at Break	Stiffness (N/m)	Work to Break (Nmm)
Ave	52,7	98	2,85	2,25	9,14	1,99	9,74	2,53	0,4874	3798	18,11
STD	3,9	25	0,21	0,17	1,90	0,21	1,80	0,28	0,0898	744	4,39

Treg Speed 2 mm/min, strip width 15 mm, gauge 20 mm, load cell 100 N

Fig. S6 Example of stress-strain curves of the keratin films prepared from the high Mw keratin fraction and plasticised with (a) 15 wt% and (b) 30 wt% glycerol concentrations.
